# Contribution of patient registries to regulatory decision making on rare diseases medicinal products in Europe

**DOI:** 10.3389/fphar.2022.924648

**Published:** 2022-08-04

**Authors:** Carla J. Jonker, Elisabeth Bakker, Xavier Kurz, Kelly Plueschke

**Affiliations:** ^1^ European Medicines Agency (EMA), Amsterdam, Netherlands; ^2^ Dutch Medicines Evaluation Board (CBG‐MEB), Utrecht, Netherlands; ^3^ Department of Clinical Pharmacy and Pharmacology, University Medical Center Groningen (UMCG), Groningen, Netherlands

**Keywords:** patient registry, orphan medicinal product (OMP), real-world evidence (RWE), guideline on registry-based studies, orphan designation

## Abstract

Between 2000 and 2021, the European Medicines Agency (EMA) assigned the orphan designation to over 1,900 medicines. Due to their small target populations, leading to challenges regarding clinical trial recruitment, study design and little knowledge on the natural history of the disease, the overall clinical evidence submitted at the time of marketing authorisation application for these medicines is often limited. Patient registries have been recognised as important sources of data on healthcare practices, drug utilisation and clinical outcomes. They may help address these challenges by providing information on epidemiology, standards of care and treatment patterns of rare diseases. In this review, we illustrate the utility of patient registries across the different stages of development of medicinal products, including orphans, to provide evidence in the context of clinical studies and to generate post-authorisation long term data on their effectiveness and safety profiles. We present important initiatives leveraging the role of registries for orphan medicinal products’ development and monitoring to ultimately improve patients’ lives.

## Introduction

Since Regulation (EC) No. 141/2000 came into force and until the end of 2021, the European Medicines Agency (EMA) assigned the orphan designation to over 1,900 medicines (European Commission Public Health, 2022). To qualify for an orphan designation, a medicine must meet a number of criteria, including the aim to treat, prevent or facilitate diagnosis of disease that is life-threatening or chronically debilitating, which prevalence in the European Union (EU) is below 5 in 10,000, or for which marketing of the medicine is unlikely to generate sufficient returns to justify the investment needed for its development. In addition, there must be no existing satisfactory method of diagnosis, prevention or treatment of the condition concerned, or, if such a method exists, the medicine must be of significant benefit to those affected by the condition (European Medicines Agency, 2018a).

In 2021, orphan medicinal products represented 26.8% of all marketing authorisation applications assessed by EMA (European Medicines Agency, 2021a). Due to the low disease prevalence, high disease severity, small and heterogeneous patient populations and limited knowledge of the disease natural history, the overall clinical evidence submitted at the time of marketing authorisation application for these medicines is often limited. Potential hurdles can be seen, for example, in clinical trials’ recruitment and designs, possibly impacted by ethical concerns of denying beneficial active treatment (Fonseca et al., 2019).

Patient registries have been recognised as potentially valuable sources of data to address these challenges and support regulatory decision-making on medicines, independent from the original purpose for which they have been established (McGettigan et al., 2019). A patient registry (hereafter referred to as registry) has been defined as “an organised system that collects uniform data (clinical and other) to identify specified outcomes for a population defined by a particular disease, condition or exposure” (Gliklich et al., 2007; European Medicines Agency, 2021b). The term “patient” highlights the focus of the registry on health information and may include patients with a certain disease, pregnant or lactating women or individuals presenting with another condition such as a birth defect or a molecular or genomic feature.

Such data source can therefore deliver useful evidence at different stages of orphan medicinal products lifecycle, including during the development phase by providing information on disease natural history, its prevalence and incidence to contextualise pre-authorisation clinical studies, to support orphan designation initial and maintenance applications by demonstrating significant benefit versus existing treatments, but also to generate post-authorisation long term data on their effectiveness and safety profiles. Orphan medicinal products are quite often granted a conditional marketing authorisation (Europe an Medicines Agency, 2016a) with specific obligations to gather comprehensive data post-approval, derived from real-world data (RWD) sources, including registries.

In this review, we outline the existing regulatory tools to integrate registries into the life cycle of a medicinal products and highlight recent studies providing real-world evidence (RWE) in regulatory submissions. Based on concrete examples, we illustrate opportunities and challenges of the use of registry data in the European Economic Area (EEA) for the assessment of medicinal products and describe some European initiatives promoting registries for regulator purposes.

## Integrating registry data in medicinal products’ life cycle

**FIGURE 1:**
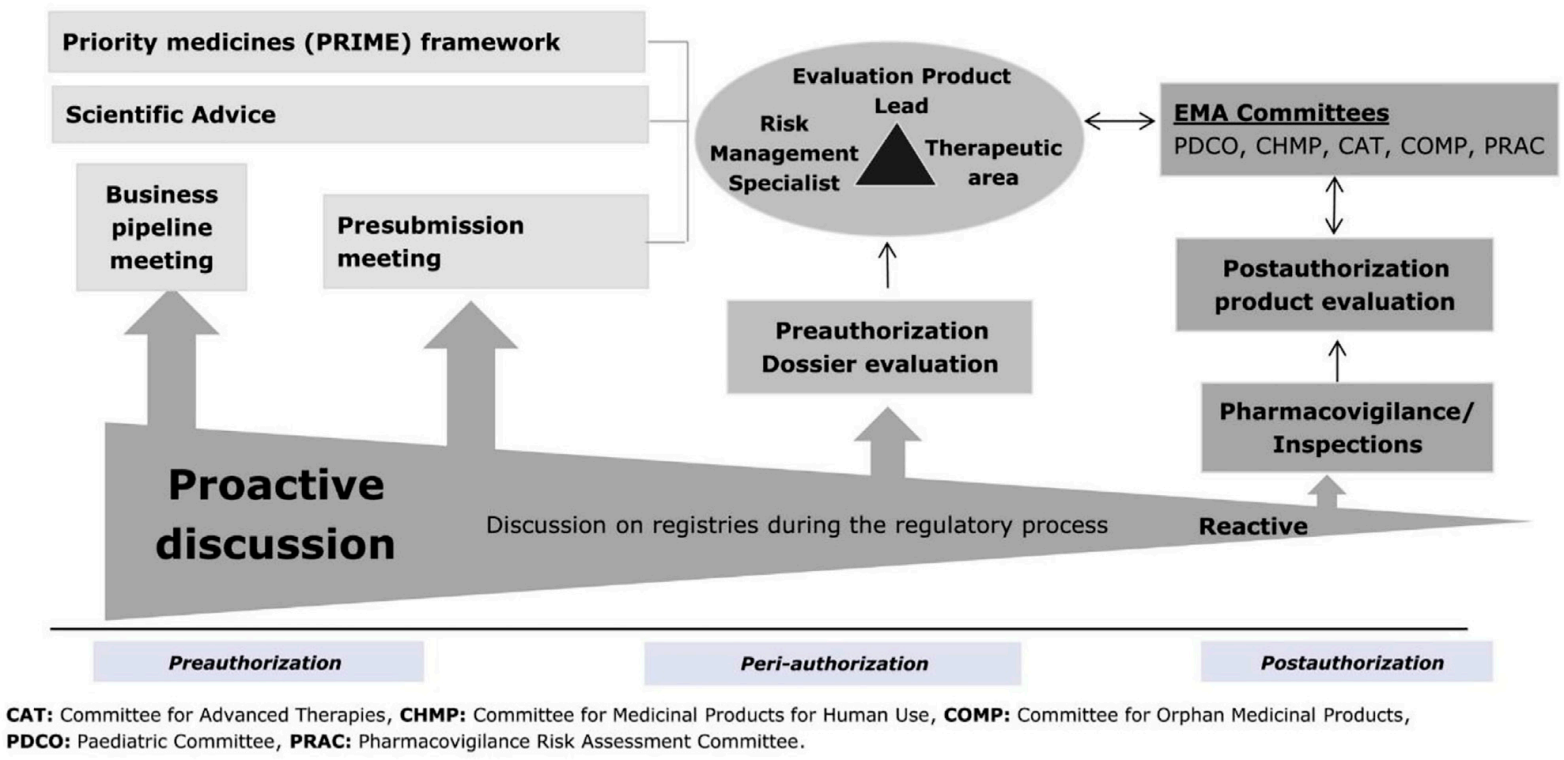
Timing of regulatory discussions on registries in the product life cycle. EMA, European Medicines Agency.

Multiple opportunities exist during products’ life cycle for proactive interactions between marketing authorisation applicants and regulators on the use of a registry, as illustrated in Figure 1 (Olmo et al., 2019). These include, for example, business pipeline meetings, innovation task force briefing meetings, kick-off meetings for PRIME products developed for an unmet medical need, dialogues during an orphan designation procedure, scientific advice procedures and pre-submission meetings (European Medicines Agency, 2000; European Medicines Agency, 2013; European Medicines Agency, 2015; European Medicines Agency, 2016b; European Medicines Agency, 2018a; European Medicines Agency, 2021c; European Medicines Agency, 2022d). Such interactions can support applicants on assessing the suitability of registries to answer specific research questions in terms of their data elements collected, data quality and governance aspects. Early engagement with registry holders is a key pre-requisite to understand the opportunities provided by registry data, but also their limitations when used to support demonstration of significant benefits of orphan medicinal products and their long-term monitoring post-authorisation. This current review focuses on the integration in later stages of development and in the context of interactions with the regulatory agencies during marketing authorisation application preparation, evaluation, and post approval monitoring.

## Increasing use of real-world evidence including registry data for regulatory purposes

Real-world evidence has been defined as the information derived from the analysis of routinely collected RWD relating to a patient’s health status or the delivery of health care from a variety of sources other than traditional clinical trials, including registries (Cave et al., 2019). Studies investigating the use of RWE for regulatory decision-making have shown that RWD is already widely used to support medicines applications. In a study where 125 dossiers for authorised orphan medicinal products published between 1999 and 2014 were reviewed, it was found that 12% did not include evidence from clinical trials but were based on literature reports, observational studies, or compassionate use programs (Pontes et al., 2018). Another study described the characteristics of RWE included in new marketing authorisation applications and extensions of indication submitted to EMA in 2018 and 2019, including orphan medicinal products. From the 158 initial marketing authorisation applications, 63 (39.9%) included RWE, out of which 38 (60.3%) were based on registries data followed by hospital data (31.7%). Registries were more frequently proposed for post-authorisation studies focusing on safety, whereas for extensions of indication, such data were also presented as evidence pre-authorisation for efficacy claims (Flynn et al., 2018). This study highlights the importance of exploiting RWE to support orphan medicinal products clinical development and post-approval monitoring, which is in line with earlier research (Eskola et al., 2019; Mahendraratnam et al., 2022; Purpura et al., 1002; Bolislis et al., 2020; Tormey et al., 2020; Feinberg et al., 2020; Sasinowski, 2012).

## Use cases demonstrating benefits of registries for regulatory purposes

### Evidence on disease natural history

Understanding the natural history of a rare disease is critical to the development of new medicinal products. This includes information on the incidence, prevalence, outcomes of the disease and characteristics of the patient population (whether untreated or treated with standards of care if any). However as highlighted above, the knowledge on rare diseases is limited for several reasons: small patient populations, spread over a broad geographically area, restricted funding to support research, complexity of disease, and delayed diagnosis (Boulanger et al., 1007). Registries can be cost-effective tools to get a grip of these scarce data. Table 1 provides details on the medicinal product ivacaftor/tezacaftor/elexacaftor (Kaftrio). During the initial marketing authorisation procedure, data from the Cystic Fibrosis Foundation Patient Registry were requested by the Committee for Medicinal Products for Human Use (CHMP) (European Medicines Agency, 2004) to illustrate the added benefit of this triple therapy to existing treatments in the subpopulation of heterozygous for *F508del* and a gating mutation (F/G) and heterozygous for *F508del* and a residual function mutation (F/RF) genotype (European Medicines Agency, 2020a). Registry data provided in the frame of the initial marketing authorisation application were not considered sufficient as the sole evidence for this subgroup. This was due to limited data quality, including lack of details on the exact modulator therapy used, the duration of therapy, specific genotypes covered and individual patient efficacy data. It was therefore considered questionable at the time whether the registry study population was sufficiently representative of the overall F/G and F/RF patients to draw conclusions on efficacy and safety in these sub-populations (European Medicines Agency, 2020). During the procedure to extend the use in patients aged 12 years and older who have at least one *F508del* mutation in the cystic fibrosis transmembrane conductance regulator (CFTR) gene, updated registry data were provided on an increased number of patients, including genotype level data on clinical end points covering both F/G and F/RF categories, which supported confirmation of a meaningful clinical benefit (European Medicines Agency, 2021d).

**TABLE 1:** Examples of products for rare diseases in which RWE was submitted in the context of marketing authorisation or extension of indication applications and to support regulatory decisions.

Product and indication	Pivotal data	Rationale for real-world evidence	Real-world data sources	Impact of RWE
**Ivacaftor/tezacaftor/elexacaftor (Kaftrio)** ^**a**^ Kaftrio is indicated in a combination regimen with ivacaftor for the treatment of cystic fibrosis (CF) in patients aged 6 years and older who have at least one *F508del* mutation in the cystic fibrosis transmembrane conductance regulator (CFTR) gene	For the extension of indication Phase 3, Randomized, Double-blind, Controlled Study Evaluating the Efficacy and Safety of Elexacaftor Combination Therapy in Subjects with CF who are heterozygous for the *F508del* mutation and a gating or residual function mutation (heterozygous for F508del and a gating mutation (F/G) and heterozygous for *F508del* and a residual function mutation (F/RF) Genotypes)	Registry data were requested by CHMP (during the initial marketing authorisation) for the subpopulation of F/G and F/RF genotypes to confirm the efficacy in this subgroup RWD from F/G and F/RF patients from the US Cystic Fibrosis Foundation Patient Registry (CFFPR) to provide post-authorisation data	US CF Foundation Patient Registry (CFFPR) • Contain data on most common disease-causing mutation • Contain data by genotype for patients who initiated treatment with ELX/TEZ/IVA	For the extension to F/RF and F/G populations the RWE analysis further confirmed the beneficial effects of ELX/TEZ/IVA in line with the effects observed in the phase 3 study Information on additional mutations were provided from the real-world effectiveness registry data Lesson learned The registry has addressed the initial concerns expressed by CHMP and adapted the data collected in order to improve the quality
**Onasemnogene abeparvovec (Zolgensma)** ^**b**^	Phase 3, open-label, single-arm, single-dose study of Zolgensma in patients with SMA Type 1 who were either symptomatic or pre-symptomatic with no functional SMN1 gene and 1 or 2 copies of SMN2 and who are <6 months (<180 days) of age at the time of gene replacement therapy (Day 1)	Need to contextualise the results of the single arm trial with evidence on natural course of disease	The PNCR natural history cohort • A natural history study of 337 patients with any form of SMA followed at 3 large, tertiary medical centres • Contain data of patients with age of onset ≤6 months, bi-allelic deletion of SMN1 and 2 copies of SMN2 • Retrospective and prospective enrolment data • Choice of primary endpoints	Comparison of single-arm trial data to a historical untreated control data showed improvement of survival after treatment with Onasemnogene that exceeds the expectations given the natural history of the disease in patients with a bi-allelic mutation in SMN1 and 2 copies of SMN2 (SMA type 1 phenotype)
Zolgensma is indicated for the treatment of: • Patients with 5q spinal muscular atrophy (SMA) with a bi-allelic mutation in the SMN1 gene and a clinical diagnosis of SMA Type 1, or • Patients with 5q SMA with a bi-allelic mutation in the SMN1 gene and up to 3 copies of the SMN2 gene	The natural history studies Paediatric Neuromuscular Clinical Research (PNCR) and NeuroNext provide the information of the natural course of the disease	Obligation to further characterise and contextualise the outcomes of SMA patients, including long-term effectiveness and safety of Zolgensma post-authorisation	NeuroNext natural history study • SMA type 1 patients with bi-allelic deletion of SMN1 and 2 copies of SMN2 were included in the comparator cohort • Contain data of SMA infants <6 months of age at 14 centres • Prospective natural history study	In order to further characterise and contextualise the outcomes of patients with a diagnosis of SMA, including long-term safety and efficacy of Zolgensma, the MAH should conduct and submit the results of a prospective observational registry study.
**Turoctocog alfa pegol (Esperoct)** ^**c**^ Treatment and prophylaxis of bleeding in patients 12 years and above with haemophilia A (congenital factor VIII deficiency)	A multi-national, multi-centre, open-label, non-controlled trial evaluating the efficacy of N8-GP for prophylaxis and treatment of bleeds in adolescent and adult patients with severe haemophilia A	Long-term safety follow-up data is needed not only to monitor development of inhibitors and allergic reactions, but also the potential effects of polyethylene glycol accumulation in the choroid plexus of the brain and other tissues/organs	European Haemophilia Safety Surveillance System (EUHASS) Registry • To collect adverse event data from turoctocog alfa pegol	The EUHASS Registry will provide data to investigate the safety of long-term exposure to turoctocog alfa pegol in patients with haemophilia A

a Kaftrio European Assessment Report (https://www.ema.europa.eu/en/documents/variation-report/kaftrio-epar-public-assessment-report-variation_en.pdf).

b Zolgensma European Assessment Report (https://www.ema.europa.eu/en/documents/assessment-report/zolgensma-epar-public-assessment-report_en.pdf).

c Esperoct European Assessment Report (https://www.ema.europa.eu/en/documents/assessment-report/esperoct-epar-public-assessment-report_en.pdf).

### Contextualisation of results of uncontrolled trials

Registries may also contextualise results of uncontrolled trials. The Society of Neuro-Oncology published an overview on the use of external control data representative of standards of care in the design and analysis of clinical trials. RWD sources presented included registries, claims and billing data, personal devices or applications, or electronic health records. As RWD is generally not collected for research purposes, there can be concerns about data organisation, data quality, potential biases (e.g., confounding factors, selection bias, etc.). The authors emphasised that high quality patient-level records, rigorous methods and validation analyses are necessary to effectively provide external data (Rahman et al., 1016).

In the case of onasemnogene abeparvovec (Zolgensma) to treat spinal muscular atrophy (SMA), two existing cohorts originating from RWD sources were used as historical control arms to contextualise results of the single arm trial (Table 1) thanks to their similarity in their patient populations’ characteristics, subtypes and endpoints/time points measurement. In addition, a registry study was imposed as a post-authorisation obligation to further characterise and contextualise the outcomes of SMA patients, including long-term safety and effectiveness of the advanced therapy medicinal product (European Medicines Agency, 2021e).

In view of the fast evolution in the clinical care management of SMA that currently counts three medicinal products (European Medicines Agency, 2017a; European Medicines Agency, 2021e; European Med icines Agency, 2021f), EMA contracted a registry-based study to investigate SMA patients’ course of disease and standards of care delivery over time. Its objective is to generate RWE on the disease progression based on the SMA types and treatments used that will support the EMA committees’ assessment, including the Committee for Advanced Therapies (European Medicines Agency, 2007), on future gene therapies developed in this field.

### Monitoring of medicines long term safety and effectiveness

Post-authorisation efficacy studies (PAES) (European Medicines Agency, 2016) and post-authorisation safety studies (PASS) (European Medicines Agency, 2017b) are usually imposed as obligation to the terms of the marketing authorisations of orphan medicinal products to gather necessary evidence on their efficacy and safety profiles once they have been released onto the market and to support regulators in their continuous assessment of these medicines’ benefit/risks balance. With the appropriate study design, registry-based PAES may assist for example in assessing the effectiveness of adapted dosing schemes applied in clinical practice (European Medicines Agency, 2016) and in studying effectiveness of medicinal products in a broader clinical disease-related context and a more heterogenous patient population (European Medicines Agency, 2021b). The conduct of post-authorisation studies is particularly important in the case of advanced therapy medicinal products (ATMPs) as these are often “first-in-class” products, for which at time of marketing authorisation the biological mechanism is not yet fully characterised, and the long-term safety is unknown. A PAES should provide confirmatory data based on use of these medicinal products in real-world clinical settings (European Medicines Agency, 2016).

Registry-based PASS could provide data to identify, characterise or quantify a safety hazard, to evaluate the safety profile of a medicinal product in long-term use (a requirement for ATMPs), to assess patterns of medicines utilisation, or to measure the effectiveness of a risk minimisation measures, e.g., by estimating its public health impact (European Medicines Agency, 2008; European Medicines Agency, 2017b). For turoctocog alfa pegol (Esperoct), a post-authorisation safety study was imposed to investigate the potential effects of polyethylene glycol accumulation in the choroid plexus of the brain and other tissues/organs (European Medicines Agency, 2020b). The European registry public health surveillance initiatives of haemophilia patients (EUHASS) is used to evaluate the longer-term safety of turoctocog alfa pegol in patients with haemophilia A and possible clinical consequences under real-world conditions of routine clinical care study (Table 1) (NN7088 -4557: Adverse Event Data Collection from the EUHASS Registry on Turoctocog alfa pegol, 2021).

For the ATMP tisagenlecleucel (Kymriah), post-authorisation studies were imposed to evaluate the long-term safety in all patients and to evaluate the efficacy and safety in all patients below the age of 3 years. Both studies are based on data from a disease registry (European Medicines Agency, 2018b).

## Challenges

Challenges exist to ensure optimised use of registries in regulatory contexts of orphan medicinal products. For example, in order to increase patient populations and statistical power of clinical studies on these medicines, data pooling from various registries and/or interoperability between these data sources are key. This requires various registries collecting data on a particular disease of interest to capture the same information according to adopted standard coding terminologies and list of common data elements, during the development of an orphan medical products as well as post-marketing. To guarantee the levels of data quality considered suitable to answer particular research questions, additional procedures may need to be put in place within registries to improve data completeness and accuracy, especially on treatments. Appropriate governance is key to clearly define data ownership, to facilitate data collection, data access, data sharing and data linkage. All these aspects can be difficult to implement due to limited funding and resources available to registries, but also due to restrictions linked to national data protection requirements (Schopohl et al., 2018; McGettigan et al., 2019). A clear sustainability plan laying down short and long terms strategies on the development and maintenance of the registries is critical to ensuring their continuous viability, adaptability and suitability to support regulators’ decision making (European Medicines Agency, 2021b).

## European initiatives promoting registries for regulatory purposes

### European Medicines Agency patient registry initiative

Various initiatives have been established to better integrate registries in research and drug development, as well as monitoring of their effects. In 2015, EMA launched the Patient Registry Initiative with the main goals to expand the use of registries for regulatory purposes (European Medicines Agency, 2018c). This provides a framework for dialogue between multi-stakeholders, including regulators, registry holders, health care professionals, patients, and medicines developers. Between 2017 and 2019, the EMA hosted five registry workshops on diseases (some of which are rare) like cystic fibrosis, multiple sclerosis, diseases for which CAR-T cell products are indicated, haemophilia, and cancers for which therapies are based on the tumours’ genetic and molecular features. These meetings brought together relevant expertise to elaborate on core data elements considered essential to be collected by registries for each particular disease in order to meet regulatory needs. Quality management processes to ensure data completeness, accuracy, and representativeness, as well as governance aspects to allow fit for purpose data access and data sharing were identified and agreed upon to support registries use in medicines benefit/risk regulatory assessments (European Medicines Agency, 2017c; European Medicines Agency, 2017d; European Medicines Agency, 2018d; European Medicines Agency, 2018e; European Medicines Agency, 2019a).

The role of registries has become more prominent in the frame of haemophilia disease following the revision of the “Guideline on the clinical investigation of recombinant and human plasma-derived Factor VIII products” (FVIII Guideline) that removed the obligation of medicines developers to perform clinical trials in previously untreated patients. Instead, post-authorisation studies are now requested for all new haemophilia medicinal products based on haemophilia registries data. The EMA workshop held in June 2018 helped explore the opportunities and challenges of using existing registries and led to the publication of a set of recommendations on utilisation of registry data in supporting regulatory evaluations of haemophilia therapies. The report also outlines actions addressed to different stakeholders to ensure use of registries is enabled accordingly, including the harmonisation of data element definitions across registries, establishment, and implementation of measures for systematic data collection with appropriate verification and quality assurance and confirm that arrangements are in place to permit data sharing (European Medicines Agency, 2018e).

### CHMP, guideline on registry-based studies

The CHMP guideline on registry-based studies was developed based on the experience gained from the aforementioned workshops, continuous dialogues with multi-stakeholders, as well as two qualification procedures on the cystic fibrosis and European Society for Blood and Marrow Transplantation registries, and the consultation procedure for the draft guideline (European Medicines Agency, 2018f; European Medicines Agency, 2018g). Following extensive consultation of regulators and health technology assessment bodies, pharmaceutical industry, registries holders, health care professionals and patients, the document was adopted by CHMP in October 2021. Its aim is to provide recommendations on key methodological aspects that are specific to the use of registries by marketing authorisation applicants and marketing authorisation holders planning to conduct registry-based studies for regulatory purposes. An annex highlights regulators’ view on good practices for the establishment and management of registries and their use for other possible regulatory purposes (European Medicines Agency, 2021b). This guideline constitutes part of the deliverables supporting implementation of the joint Heads of Medicines Agencies/EMA Big Data Steering Group recommendations to support data driven, evidence-based, robust decision-making on medicinal products (European Medicines Agency, 2022a).

In parallel, the European Network of Health Technology Assessment developed the Registry Evaluation and Quality Standards (REQueST) tool to improve the quality of registries and to support consistent evaluation of the suitability of such data sources by Health Technology Assessment in the context of treatments reimbursement (European Network of Health Technology Assessment, 2021).

### Big data framework

EMA has outlined its vision that by 2025 the use of real-world evidence will have been enabled and its value will have been established across the spectrum of regulatory use cases (Kjaer et al., 2022). To achieve this goal, an EU-wide federated network of data, expertise, and services namely the Data Analytics and Real-World Interrogation Network (DARWIN EU) has been established (European Medicines Agency, 2021g). DARWIN EU will support regulatory decision-making by establishing and maintaining a catalogue of known, relevant data holders, continually ensuring the discoverability and quality of data held by data holders in order to conduct scientific studies and analyses on behalf of the European Medicines Regulatory Network and EMA scientific committees. The idea is to continuously expand the catalogue to new data sources, such as registries, once conforming with required standards for usage in regulatory context. DARWIN EU will facilitate assessments by the Committee for Orphan Medicinal Products (COMP) of orphan designation requests by performing studies on disease prevalence and incidence, and/or on their standards of care to confirm or refute applicants’ applications. It will also support other committees through the conduct of drug utilisation studies, or studies looking at the long-term safety and effectiveness profiles of orphan medicinal products post-approval.

### European Reference Networks

European Reference Networks (ERNs) are virtual networks of healthcare providers across Europe (24 as of April 2022) that aim to facilitate discussions on complex or rare diseases and conditions that require highly specialised treatment, and concentrated knowledge and resources [European Reference Networks (ERNs), 2021]. Each ERN has leveraged or developed registries for research purposes on rare diseases thanks to financial support from the European Commission. To build on the strength of the individual ERNs and to create a platform that integrates all ERNs research and innovation capacity, the European Rare Disease Research Coordination and Support Action consortium (ERICA) has been created. One work focus of ERICA is on coordinated activities to advance the development and integration of ERN-wide rare disease registries and their utilisation for joint research initiatives. The idea is to facilitate the access of data in accordance with the Findable, Accessible, Interoperable and Reusable (FAIR) principles whilst complying with data protection requirements (ERICA, 2022). The agreed harmonised set of 16 common data elements is published on the European platform on rare disease registration (EU RD Platform) (European Commission, 2022a). These will be collected by all ERN registries to allow standardisation and potential linkage between the data sources while the registries remain the owners of their data European Rare Disease Registry Infrastructure (ERDRI) (ERDRI European Rare Disease Registry Infrastructure, 2022). Although, details on the use of any medical products are not (yet) part of this common data set, ERN registries are already part of medicines regulatory lifecycle, for example the EURACAN registry that is being used to collect long term data on larotrectinib (Vitrakvi), a cancer medicine for treating solid tumours that display a neurotrophic tyrosine receptor kinase (NTRK) gene fusion. These tumours produce an abnormal protein (TRK fusion protein), which causes the uncontrolled growth of cancer cells. The EURACAN registry will support the characterisation of the safety profile of larotrectinib, in particular to address severe neurologic reactions, severe drug-induced liver injury, serious infections secondary to neutropenia and use in pregnancy and lactation. These data will be provided *via* annual summary reports from the EURACAN registry (European Medicines Agency, 2019b; EURACAN registry, 2022). Another example is the European Rare Kidney Disease Registry (ERKReg), where medication-related information is prospectively captured in disease-specific sub registries, e.g., for systemic lupus erythematosus nephritis and cystinuria (Bassanese et al., 2021). Ideally, the ERN registries will be 1 day integrated into DARWIN EU and be part of the European Health Data Space (European Commission, 2022b).

Besides health care professional-driven initiatives, some patient-led initiatives or patient empowered registries aim to being trial-ready such as the Duchenne Data Foundation and the Italian Neuromuscular Registry (Ambrosini et al., 2018; van Lin et al., 2021). This registry illustrates that patient registries have several purposes, such as to monitor the clinical status, quality of life, comorbidities, and treatments of patients over time or to monitor and improve overall quality of care (European Medicines Agency, 2021b). All these initiatives account for the same objective which is to make data more rapidly accessible for research for new treatments.

### Continuous collaboration with international regulatory partners

EMA holds regular meetings with other non-EU regulators in so-called “clusters” (European Medicines Agency, 2022b). The cluster on rare diseases has for objective to facilitate exchange of information on the development and scientific evaluation of medicines for rare diseases (European Medicines Agency, 2022c). This includes conducting clinical trials in small populations, obtaining preclinical evidence to support development programmes, risk management strategies for long-term safety issues and the design of post-marketing studies, in particular in the context of early access mechanisms such as EMA’s conditional marketing authorisation and Food and Drug Administration’s accelerated approval (Food and Drug Administration, 2020). This cluster complements the one on orphan medicinal products, which mainly focuses on orphan designation.

Finally, a newly created cluster focuses on the use of RWE in the development and monitoring of medicinal products including those intended for rare diseases. The idea is to share experience and best practice on regulatory assessment of RWE, including that based on registries data, submitted as part of medicines applications. This cluster creates a bridge across regulatory agencies worldwide that supports dialogue and alignment on how to best integrate real-world (including registries) into medicines lifecycle in view to promote faster access to innovative, safe and effective treatments (European Medicines Agency, 2014).

## Conclusion

We have demonstrated that registries can be used for several purposes in view to support regulatory decisions on orphan medicinal products. They can fill knowledge gaps by providing natural history data, information on standard of care, by creating historical comparator cohort/external control arm in the frame of clinical trials, and by providing long term safety and effectiveness data. The suitability of using registries needs to be assessed on a case-by-case basis, carefully balancing what opportunities they bring over their possible limitations, as to the data elements captured, the data quality and important governance aspects. For this reason, the EMA Guideline on registry-based studies recommends performing a feasibility analysis and a quality management to ensure data integrity, completeness, and security. At an early-stage collaboration between pharmaceutical companies and registry holders will help to understand the suitability of the registry to answer a specific research question (European Medicines Agency, 2021b). The multiple initiatives launched at European and international levels will provide frameworks to promote the values of registries from all stakeholders’ perspectives. Early and continuous dialogue facilitates sharing of experience and best practice to help further improve the quality of registries and allow their full exploitation in medicines research, development and monitoring for faster patients access to innovative treatments.
